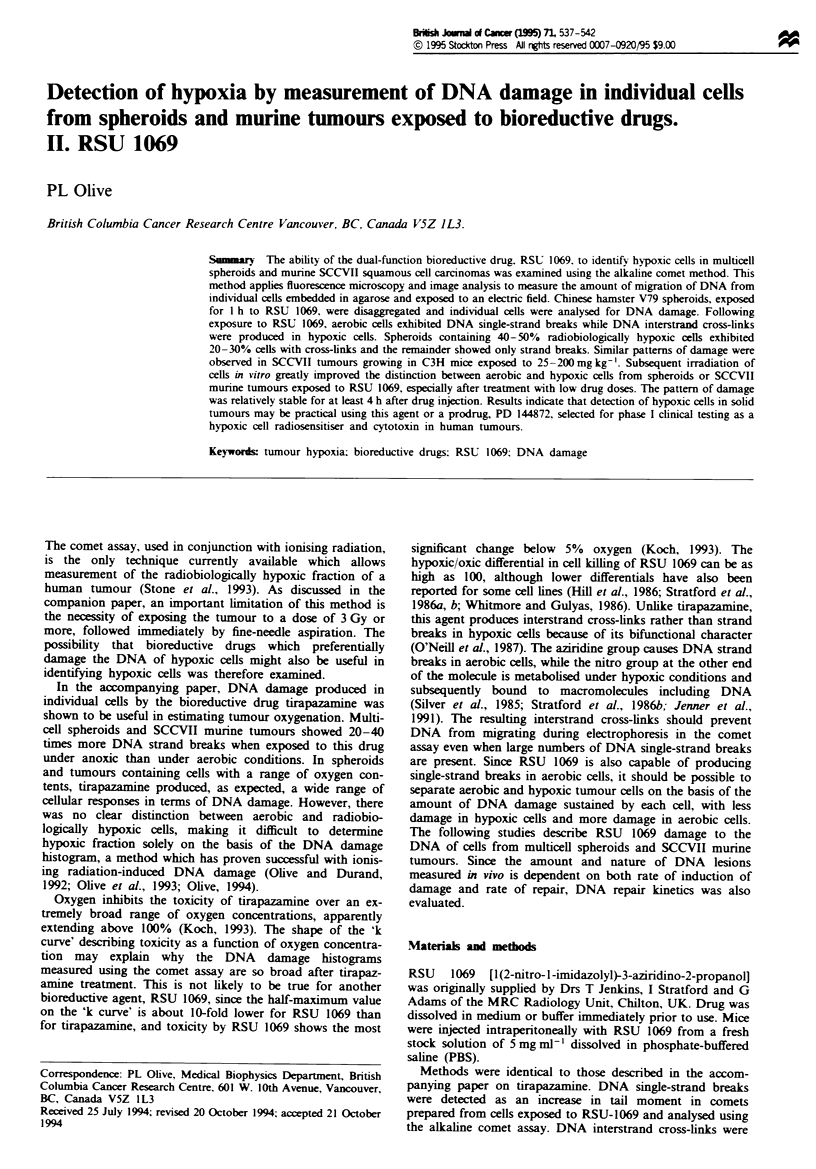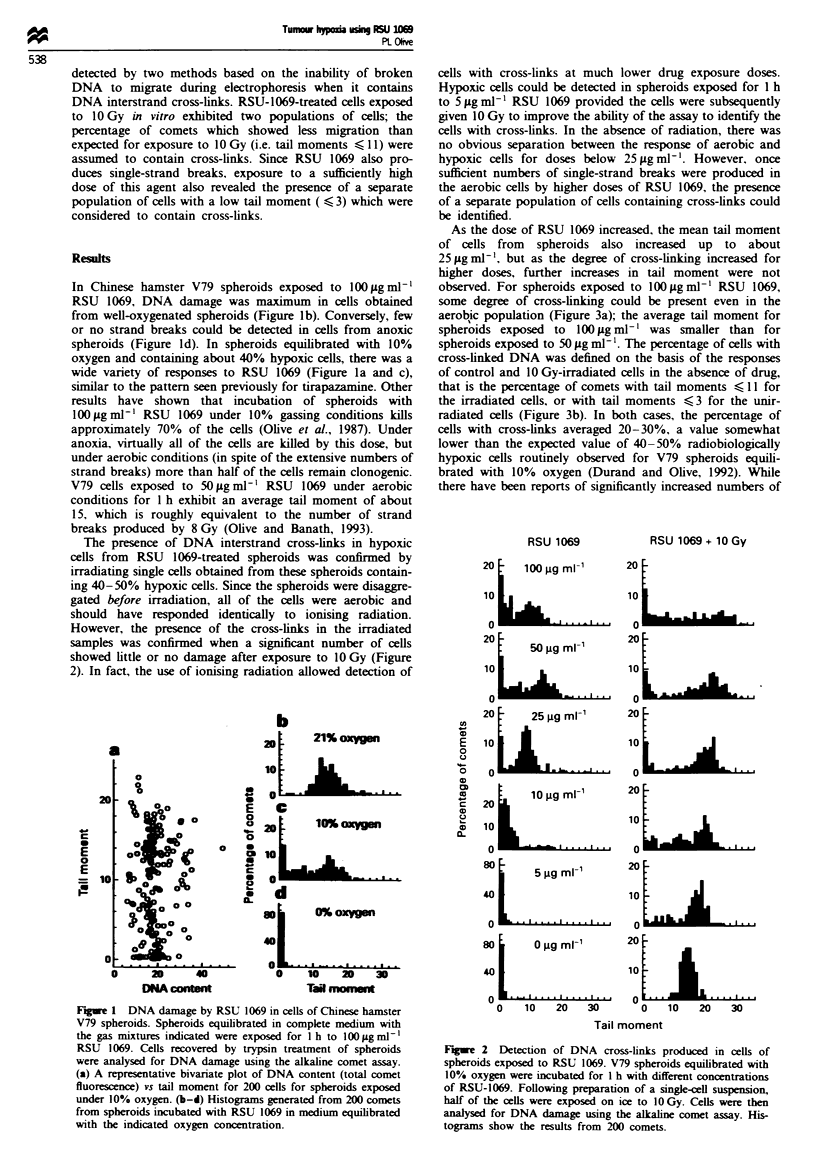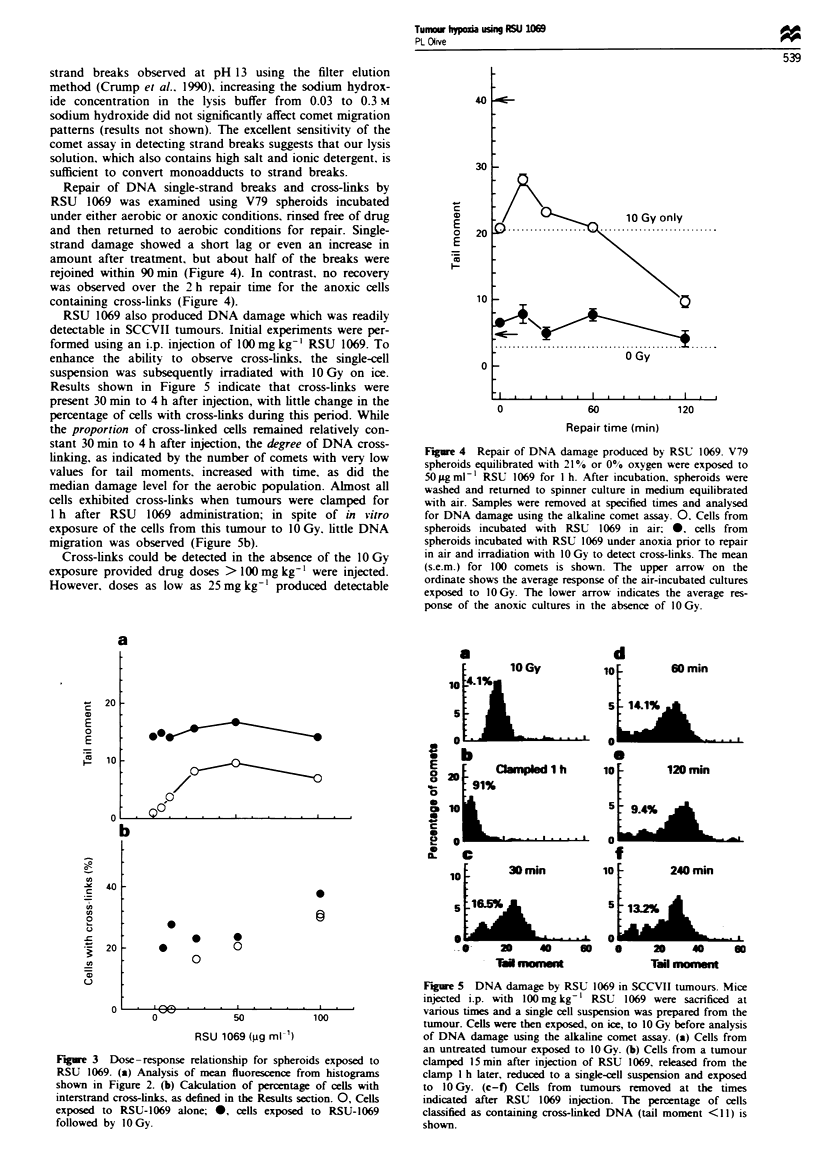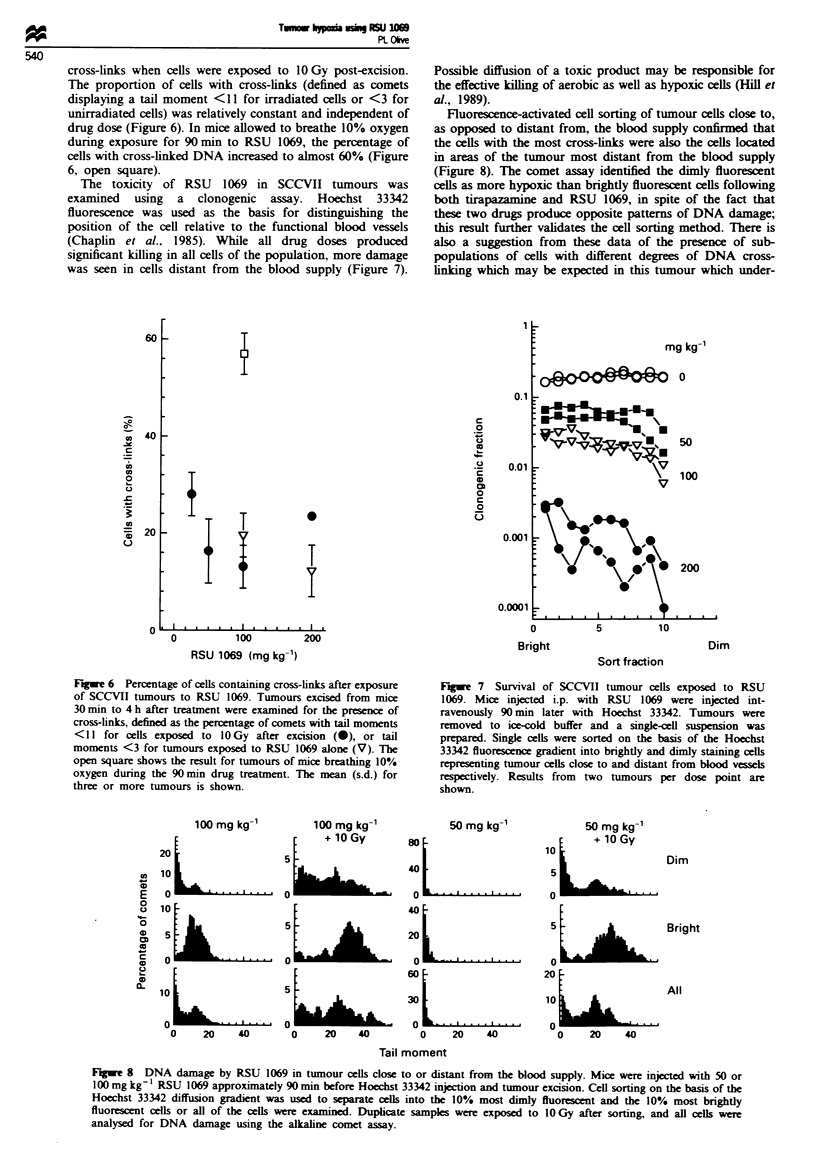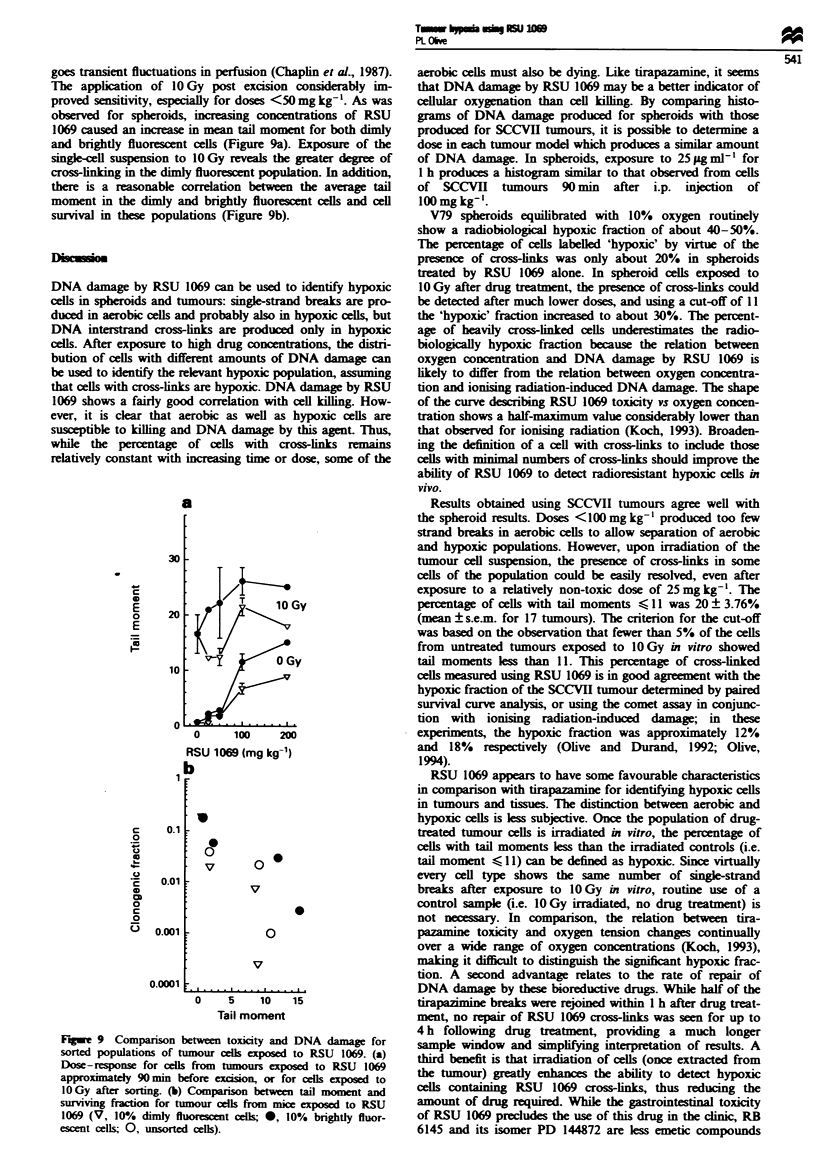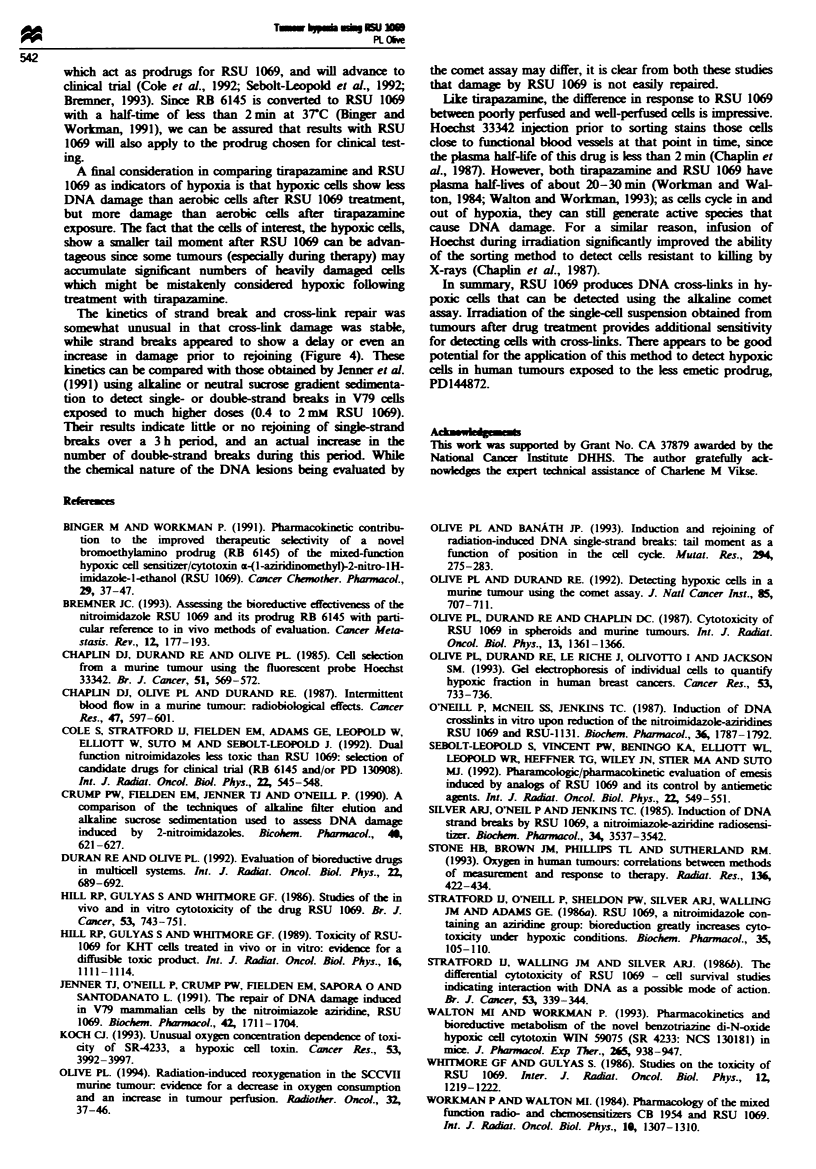# Detection of hypoxia by measurement of DNA damage in individual cells from spheroids and murine tumours exposed to bioreductive drugs. II. RSU 1069.

**DOI:** 10.1038/bjc.1995.106

**Published:** 1995-03

**Authors:** P. L. Olive

**Affiliations:** British Columbia Cancer Research Centre Vancouver, Canada.

## Abstract

The ability of the dual-function bioreductive drug, RSU 1069, to identify hypoxic cells in multicell spheroids and murine SCCVII squamous cell carcinomas was examined using the alkaline comet method. This method applies fluorescence microscopy and image analysis to measure the amount of migration of DNA from individual cells embedded in agarose and exposed to an electric field. Chinese hamster V79 spheroids, exposed for 1 h to RSU 1069, were disaggregated and individual cells were analysed for DNA damage. Following exposure to RSU 1069, aerobic cells exhibited DNA single-strand breaks while DNA interstrand cross-links were produced in hypoxic cells. Spheroids containing 40-50% radiobiologically hypoxic cells exhibited 20-30% cells with cross-links and the remainder showed only strand breaks. Similar patterns of damage were observed in SCCVII tumours growing in C3H mice exposed to 25-200 mg kg-1. Subsequent irradiation of cells in vitro greatly improved the distinction between aerobic and hypoxic cells from spheroids or SCCVII murine tumours exposed to RSU 1069, especially after treatment with low drug doses. The pattern of damage was relatively stable for at least 4 h after drug injection. Results indicate that detection of hypoxic cells in solid tumours may be practical using this agent or a prodrug, PD 144872, selected for phase I clinical testing as a hypoxic cell radiosensitiser and cytotoxin in human tumours.


British Journal of Cancer (1995) 71, 537-542

© 1995 Stockton Press All rights reserved 0007-0920/95 $9.00

### Detection of hypoxia by measurement of DNA damage in individual cells from spheroids and murine tumours exposed to bioreductive drugs. II. RSU 1069

PL Olive

*British Columbia Cancer Research Centre Vancouver, BC, Canada V5Z 1L3.*

**Summary** The ability of the dual-function bioreductive drug, RSU 1069, to identify hypoxic cells in multicell spheroids and murine SCCVII squamous cell carcinomas was examined using the alkaline comet method. This method applies fluorescence microscopy and image analysis to measure the amount of migration of DNA from individual cells embedded in agarose and exposed to an electric field. Chinese hamster V79 spheroids, exposed for 1 h to RSU 1069, were disaggregated and individual cells were analysed for DNA damage. Following exposure to RSU 1069, aerobic cells exhibited DNA single-strand breaks while DNA interstrand cross-links were produced in hypoxic cells. Spheroids containing 40-50% radiobiologically hypoxic cells exhibited 20-30% cells with cross-links and the remainder showed only strand breaks. Similar patterns of damage were observed in SCCVII tumours growing in C3H mice exposed to 25-200 mg $kg^{-1}$. Subsequent irradiation of cells *in vitro* greatly improved the distinction between aerobic and hypoxic cells from spheroids or SCCVII murine tumours exposed to RSU 1069, especially after treatment with low drug doses. The pattern of damage was relatively stable for at least 4 h after drug injection. Results indicate that detection of hypoxic cells in solid tumours may be practical using this agent or a prodrug, PD 144872, selected for phase I clinical testing as a hypoxic cell radiosensitiser and cytotoxin in human tumours.

**Keywords:** tumour hypoxia; bioreductive drugs; RSU 1069; DNA damage

The comet assay, used in conjunction with ionising radiation, is the only technique currently available which allows measurement of the radiobiologically hypoxic fraction of a human tumour (Stone *et al.*, 1993). As discussed in the companion paper, an important limitation of this method is the necessity of exposing the tumour to a dose of 3 Gy or more, followed immediately by fine-needle aspiration. The possibility that bioreductive drugs which preferentially damage the DNA of hypoxic cells might also be useful in identifying hypoxic cells was therefore examined.

In the accompanying paper, DNA damage produced in individual cells by the bioreductive drug tirapazamine was shown to be useful in estimating tumour oxygenation. Multicell spheroids and SCCVII murine tumours showed 20-40 times more DNA strand breaks when exposed to this drug under anoxic than under aerobic conditions. In spheroids and tumours containing cells with a range of oxygen contents, tirapazamine produced, as expected, a wide range of cellular responses in terms of DNA damage. However, there was no clear distinction between aerobic and radiobiologically hypoxic cells, making it difficult to determine hypoxic fraction solely on the basis of the DNA damage histogram, a method which has proven successful with ionising radiation-induced DNA damage (Olive and Durand, 1992; Olive *et al.*, 1993; Olive, 1994).

Oxygen inhibits the toxicity of tirapazamine over an extremely broad range of oxygen concentrations, apparently extending above 100% (Koch, 1993). The shape of the 'k curve' describing toxicity as a function of oxygen concentration may explain why the DNA damage histograms measured using the comet assay are so broad after tirapazamine treatment. This is not likely to be true for another bioreductive agent, RSU 1069, since the half-maximum value on the 'k curve' is about 10-fold lower for RSU 1069 than for tirapazamine, and toxicity by RSU 1069 shows the most significant change below 5% oxygen (Koch, 1993). The hypoxic/oxic differential in cell killing of RSU 1069 can be as high as 100, although lower differentials have also been reported for some cell lines (Hill *et al.*, 1986; Stratford *et al.*, 1986*a*, *b*; Whitmore and Gulyas, 1986). Unlike tirapazamine, this agent produces interstrand cross-links rather than strand breaks in hypoxic cells because of its bifunctional character (O'Neill *et al.*, 1987). The aziridine group causes DNA strand breaks in aerobic cells, while the nitro group at the other end of the molecule is metabolised under hypoxic conditions and subsequently bound to macromolecules including DNA (Silver *et al.*, 1985; Stratford *et al.*, 1986*b*; *Jenner et al.*, 1991). The resulting interstrand cross-links should prevent DNA from migrating during electrophoresis in the comet assay even when large numbers of DNA single-strand breaks are present. Since RSU 1069 is also capable of producing single-strand breaks in aerobic cells, it should be possible to separate aerobic and hypoxic tumour cells on the basis of the amount of DNA damage sustained by each cell, with less damage in hypoxic cells and more damage in aerobic cells. The following studies describe RSU 1069 damage to the DNA of cells from multicell spheroids and SCCVII murine tumours. Since the amount and nature of DNA lesions measured *in vivo* is dependent on both rate of induction of damage and rate of repair, DNA repair kinetics was also evaluated.

### Materials and methods

RSU 1069 [1(2-nitro-1-imidazolyl)-3-aziridino-2-propanol] was originally supplied by Drs T Jenkins, I Stratford and G Adams of the MRC Radiology Unit, Chilton, UK. Drug was dissolved in medium or buffer immediately prior to use. Mice were injected intraperitoneally with RSU 1069 from a fresh stock solution of 5 mg $ml^{-1}$ dissolved in phosphate-buffered saline (PBS).

Methods were identical to those described in the accompanying paper on tirapazamine. DNA single-strand breaks were detected as an increase in tail moment in comets prepared from cells exposed to RSU-1069 and analysed using the alkaline comet assay. DNA interstrand cross-links were

Correspondence: PL Olive, Medical Biophysics Department, British Columbia Cancer Research Centre, 601 W. 10th Avenue, Vancouver, BC, Canada V5Z 1L3
Received 25 July 1994; revised 20 October 1994; accepted 21 October 1994

detected by two methods based on the inability of broken DNA to migrate during electrophoresis when it contains DNA interstrand cross-links. RSU-1069-treated cells exposed to 10 Gy *in vitro* exhibited two populations of cells; the percentage of comets which showed less migration than expected for exposure to 10 Gy (i.e. tail moments $\leqslant 11$) were assumed to contain cross-links. Since RSU 1069 also produces single-strand breaks, exposure to a sufficiently high dose of this agent also revealed the presence of a separate population of cells with a low tail moment ($\leqslant 3$) which were considered to contain cross-links.

### Results

In Chinese hamster V79 spheroids exposed to $100\,\mu g\,ml^{-1}$ RSU 1069, DNA damage was maximum in cells obtained from well-oxygenated spheroids (Figure 1b). Conversely, few or no strand breaks could be detected in cells from anoxic spheroids (Figure 1d). In spheroids equilibrated with 10% oxygen and containing about 40% hypoxic cells, there was a wide variety of responses to RSU 1069 (Figure 1a and c), similar to the pattern seen previously for tirapazamine. Other results have shown that incubation of spheroids with $100\,\mu g\,ml^{-1}$ RSU 1069 under 10% gassing conditions kills approximately 70% of the cells (Olive *et al.*, 1987). Under anoxia, virtually all of the cells are killed by this dose, but under aerobic conditions (in spite of the extensive numbers of strand breaks) more than half of the cells remain clonogenic. V79 cells exposed to $50\,\mu g\,ml^{-1}$ RSU 1069 under aerobic conditions for 1 h exhibit an average tail moment of about 15, which is roughly equivalent to the number of strand breaks produced by 8 Gy (Olive and Banath, 1993).

The presence of DNA interstrand cross-links in hypoxic cells from RSU 1069-treated spheroids was confirmed by irradiating single cells obtained from these spheroids containing 40–50% hypoxic cells. Since the spheroids were disaggregated *before* irradiation, all of the cells were aerobic and should have responded identically to ionising radiation. However, the presence of the cross-links in the irradiated samples was confirmed when a significant number of cells showed little or no damage after exposure to 10 Gy (Figure 2). In fact, the use of ionising radiation allowed detection of cells with cross-links at much lower drug exposure doses. Hypoxic cells could be detected in spheroids exposed for 1 h to $5\,\mu g\,ml^{-1}$ RSU 1069 provided the cells were subsequently given 10 Gy to improve the ability of the assay to identify the cells with cross-links. In the absence of radiation, there was no obvious separation between the response of aerobic and hypoxic cells for doses below $25\,\mu g\,ml^{-1}$. However, once sufficient numbers of single-strand breaks were produced in the aerobic cells by higher doses of RSU 1069, the presence of a separate population of cells containing cross-links could be identified.

As the dose of RSU 1069 increased, the mean tail moment of cells from spheroids also increased up to about $25\,\mu g\,ml^{-1}$, but as the degree of cross-linking increased for higher doses, further increases in tail moment were not observed. For spheroids exposed to $100\,\mu g\,ml^{-1}$ RSU 1069, some degree of cross-linking could be present even in the aerobic population (Figure 3a); the average tail moment for spheroids exposed to $100\,\mu g\,ml^{-1}$ was smaller than for spheroids exposed to $50\,\mu g\,ml^{-1}$. The percentage of cells with cross-linked DNA was defined on the basis of the responses of control and 10 Gy-irradiated cells in the absence of drug, that is the percentage of comets with tail moments $\leqslant 11$ for the irradiated cells, or with tail moments $\leqslant 3$ for the unirradiated cells (Figure 3b). In both cases, the percentage of cells with cross-links averaged 20–30%, a value somewhat lower than the expected value of 40–50% radiobiologically hypoxic cells routinely observed for V79 spheroids equilibrated with 10% oxygen (Durand and Olive, 1992). While there have been reports of significantly increased numbers of

**Figure 1** DNA damage by RSU 1069 in cells of Chinese hamster V79 spheroids. Spheroids equilibrated in complete medium with the gas mixtures indicated were exposed for 1 h to $100\,\mu g\,ml^{-1}$ RSU 1069. Cells recovered by trypsin treatment of spheroids were analysed for DNA damage using the alkaline comet assay. (**a**) A representative bivariate plot of DNA content (total comet fluorescence) *vs* tail moment for 200 cells for spheroids exposed under 10% oxygen. (**b**–**d**) Histograms generated from 200 comets from spheroids incubated with RSU 1069 in medium equilibrated with the indicated oxygen concentration.

**Figure 2** Detection of DNA cross-links produced in cells of spheroids exposed to RSU 1069. V79 spheroids equilibrated with 10% oxygen were incubated for 1 h with different concentrations of RSU-1069. Following preparation of a single-cell suspension, half of the cells were exposed on ice to 10 Gy. Cells were then analysed for DNA damage using the alkaline comet assay. Histograms show the results from 200 comets.

strand breaks observed at pH 13 using the filter elution method (Crump *et al.*, 1990), increasing the sodium hydroxide concentration in the lysis buffer from 0.03 to 0.3 M sodium hydroxide did not significantly affect comet migration patterns (results not shown). The excellent sensitivity of the comet assay in detecting strand breaks suggests that our lysis solution, which also contains high salt and ionic detergent, is sufficient to convert monoadducts to strand breaks.

Repair of DNA single-strand breaks and cross-links by RSU 1069 was examined using V79 spheroids incubated under either aerobic or anoxic conditions, rinsed free of drug and then returned to aerobic conditions for repair. Single-strand damage showed a short lag or even an increase in amount after treatment, but about half of the breaks were rejoined within 90 min (Figure 4). In contrast, no recovery was observed over the 2 h repair time for the anoxic cells containing cross-links (Figure 4).

RSU 1069 also produced DNA damage which was readily detectable in SCCVII tumours. Initial experiments were performed using an i.p. injection of 100 mg $kg^{-1}$ RSU 1069. To enhance the ability to observe cross-links, the single-cell suspension was subsequently irradiated with 10 Gy on ice. Results shown in Figure 5 indicate that cross-links were present 30 min to 4 h after injection, with little change in the percentage of cells with cross-links during this period. While the *proportion* of cross-linked cells remained relatively constant 30 min to 4 h after injection, the *degree* of DNA cross-linking, as indicated by the number of comets with very low values for tail moments, increased with time, as did the median damage level for the aerobic population. Almost all cells exhibited cross-links when tumours were clamped for 1 h after RSU 1069 administration; in spite of *in vitro* exposure of the cells from this tumour to 10 Gy, little DNA migration was observed (Figure 5b).

Cross-links could be detected in the absence of the 10 Gy exposure provided drug doses $>100$ mg $kg^{-1}$ were injected. However, doses as low as 25 mg $kg^{-1}$ produced detectable

**Figure 4** Repair of DNA damage produced by RSU 1069. V79 spheroids equilibrated with 21% or 0% oxygen were exposed to 50 μg $ml^{-1}$ RSU 1069 for 1 h. After incubation, spheroids were washed and returned to spinner culture in medium equilibrated with air. Samples were removed at specified times and analysed for DNA damage using the alkaline comet assay. ○, Cells from spheroids incubated with RSU 1069 in air; ●, cells from spheroids incubated with RSU 1069 under anoxia prior to repair in air and irradiation with 10 Gy to detect cross-links. The mean (s.e.m.) for 100 comets is shown. The upper arrow on the ordinate shows the average response of the air-incubated cultures exposed to 10 Gy. The lower arrow indicates the average response of the anoxic cultures in the absence of 10 Gy.

**Figure 3** Dose-response relationship for spheroids exposed to RSU 1069. (**a**) Analysis of mean fluorescence from histograms shown in Figure 2. (**b**) Calculation of percentage of cells with interstrand cross-links, as defined in the Results section. ○, Cells exposed to RSU-1069 alone; ●, cells exposed to RSU-1069 followed by 10 Gy.

**Figure 5** DNA damage by RSU 1069 in SCCVII tumours. Mice injected i.p. with 100 mg $kg^{-1}$ RSU 1069 were sacrificed at various times and a single cell suspension was prepared from the tumour. Cells were then exposed, on ice, to 10 Gy before analysis of DNA damage using the alkaline comet assay. (**a**) Cells from an untreated tumour exposed to 10 Gy. (**b**) Cells from a tumour clamped 15 min after injection of RSU 1069, released from the clamp 1 h later, reduced to a single-cell suspension and exposed to 10 Gy. (**c**–**f**) Cells from tumours removed at the times indicated after RSU 1069 injection. The percentage of cells classified as containing cross-linked DNA (tail moment $<11$) is shown.

cross-links when cells were exposed to 10 Gy post-excision. The proportion of cells with cross-links (defined as comets displaying a tail moment <11 for irradiated cells or <3 for unirradiated cells) was relatively constant and independent of drug dose (Figure 6). In mice allowed to breathe 10% oxygen during exposure for 90 min to RSU 1069, the percentage of cells with cross-linked DNA increased to almost 60% (Figure 6, open square).

The toxicity of RSU 1069 in SCCVII tumours was examined using a clonogenic assay. Hoechst 33342 fluorescence was used as the basis for distinguishing the position of the cell relative to the functional blood vessels (Chaplin *et al.*, 1985). While all drug doses produced significant killing in all cells of the population, more damage was seen in cells distant from the blood supply (Figure 7). Possible diffusion of a toxic product may be responsible for the effective killing of aerobic as well as hypoxic cells (Hill *et al.*, 1989).

Fluorescence-activated cell sorting of tumour cells close to, as opposed to distant from, the blood supply confirmed that the cells with the most cross-links were also the cells located in areas of the tumour most distant from the blood supply (Figure 8). The comet assay identified the dimly fluorescent cells as more hypoxic than brightly fluorescent cells following both tirapazamine and RSU 1069, in spite of the fact that these two drugs produce opposite patterns of DNA damage; this result further validates the cell sorting method. There is also a suggestion from these data of the presence of subpopulations of cells with different degrees of DNA cross-linking which may be expected in this tumour which under-

**Figure 6** Percentage of cells containing cross-links after exposure of SCCVII tumours to RSU 1069. Tumours excised from mice 30 min to 4 h after treatment were examined for the presence of cross-links, defined as the percentage of comets with tail moments <11 for cells exposed to 10 Gy after excision (●), or tail moments <3 for tumours exposed to RSU 1069 alone (▽). The open square shows the result for tumours of mice breathing 10% oxygen during the 90 min drug treatment. The mean (s.d.) for three or more tumours is shown.

**Figure 7** Survival of SCCVII tumour cells exposed to RSU 1069. Mice injected i.p. with RSU 1069 were injected intravenously 90 min later with Hoechst 33342. Tumours were removed to ice-cold buffer and a single-cell suspension was prepared. Single cells were sorted on the basis of the Hoechst 33342 fluorescence gradient into brightly and dimly staining cells representing tumour cells close to and distant from blood vessels respectively. Results from two tumours per dose point are shown.

**Figure 8** DNA damage by RSU 1069 in tumour cells close to or distant from the blood supply. Mice were injected with 50 or 100 mg kg$^{-1}$ RSU 1069 approximately 90 min before Hoechst 33342 injection and tumour excision. Cell sorting on the basis of the Hoechst 33342 diffusion gradient was used to separate cells into the 10% most dimly fluorescent and the 10% most brightly fluorescent cells or all of the cells were examined. Duplicate samples were exposed to 10 Gy after sorting, and all cells were analysed for DNA damage using the alkaline comet assay.

goes transient fluctuations in perfusion (Chaplin *et al.*, 1987). The application of 10 Gy post excision considerably improved sensitivity, especially for doses <50 mg kg$^{-1}$. As was observed for spheroids, increasing concentrations of RSU 1069 caused an increase in mean tail moment for both dimly and brightly fluorescent cells (Figure 9a). Exposure of the single-cell suspension to 10 Gy reveals the greater degree of cross-linking in the dimly fluorescent population. In addition, there is a reasonable correlation between the average tail moment in the dimly and brightly fluorescent cells and cell survival in these populations (Figure 9b).

### Discussion

DNA damage by RSU 1069 can be used to identify hypoxic cells in spheroids and tumours: single-strand breaks are produced in aerobic cells and probably also in hypoxic cells, but DNA interstrand cross-links are produced only in hypoxic cells. After exposure to high drug concentrations, the distribution of cells with different amounts of DNA damage can be used to identify the relevant hypoxic population, assuming that cells with cross-links are hypoxic. DNA damage by RSU 1069 shows a fairly good correlation with cell killing. However, it is clear that aerobic as well as hypoxic cells are susceptible to killing and DNA damage by this agent. Thus, while the percentage of cells with cross-links remains relatively constant with increasing time or dose, some of the

**Figure 9** Comparison between toxicity and DNA damage for sorted populations of tumour cells exposed to RSU 1069. (**a**) Dose–response for cells from tumours exposed to RSU 1069 approximately 90 min before excision, or for cells exposed to 10 Gy after sorting. (**b**) Comparison between tail moment and surviving fraction for tumour cells from mice exposed to RSU 1069 (∇, 10% dimly fluorescent cells; ●, 10% brightly fluorescent cells; ○, unsorted cells).

aerobic cells must also be dying. Like tirapazamine, it seems that DNA damage by RSU 1069 may be a better indicator of cellular oxygenation than cell killing. By comparing histograms of DNA damage produced for spheroids with those produced for SCCVII tumours, it is possible to determine a dose in each tumour model which produces a similar amount of DNA damage. In spheroids, exposure to 25 $\mu$g ml$^{-1}$ for 1 h produces a histogram similar to that observed from cells of SCCVII tumours 90 min after i.p. injection of 100 mg kg$^{-1}$.

V79 spheroids equilibrated with 10% oxygen routinely show a radiobiological hypoxic fraction of about 40–50%. The percentage of cells labelled 'hypoxic' by virtue of the presence of cross-links was only about 20% in spheroids treated by RSU 1069 alone. In spheroid cells exposed to 10 Gy after drug treatment, the presence of cross-links could be detected after much lower doses, and using a cut-off of 11 the 'hypoxic' fraction increased to about 30%. The percentage of heavily cross-linked cells underestimates the radiobiologically hypoxic fraction because the relation between oxygen concentration and DNA damage by RSU 1069 is likely to differ from the relation between oxygen concentration and ionising radiation-induced DNA damage. The shape of the curve describing RSU 1069 toxicity *vs* oxygen concentration shows a half-maximum value considerably lower than that observed for ionising radiation (Koch, 1993). Broadening the definition of a cell with cross-links to include those cells with minimal numbers of cross-links should improve the ability of RSU 1069 to detect radioresistant hypoxic cells *in vivo*.

Results obtained using SCCVII tumours agree well with the spheroid results. Doses <100 mg kg$^{-1}$ produced too few strand breaks in aerobic cells to allow separation of aerobic and hypoxic populations. However, upon irradiation of the tumour cell suspension, the presence of cross-links in some cells of the population could be easily resolved, even after exposure to a relatively non-toxic dose of 25 mg kg$^{-1}$. The percentage of cells with tail moments $\leqslant 11$ was $20 \pm 3.76$% (mean $\pm$ s.e.m. for 17 tumours). The criterion for the cut-off was based on the observation that fewer than 5% of the cells from untreated tumours exposed to 10 Gy *in vitro* showed tail moments less than 11. This percentage of cross-linked cells measured using RSU 1069 is in good agreement with the hypoxic fraction of the SCCVII tumour determined by paired survival curve analysis, or using the comet assay in conjunction with ionising radiation-induced damage; in these experiments, the hypoxic fraction was approximately 12% and 18% respectively (Olive and Durand, 1992; Olive, 1994).

RSU 1069 appears to have some favourable characteristics in comparison with tirapazamine for identifying hypoxic cells in tumours and tissues. The distinction between aerobic and hypoxic cells is less subjective. Once the population of drug-treated tumour cells is irradiated *in vitro*, the percentage of cells with tail moments less than the irradiated controls (i.e. tail moment $\leqslant 11$) can be defined as hypoxic. Since virtually every cell type shows the same number of single-strand breaks after exposure to 10 Gy *in vitro*, routine use of a control sample (i.e. 10 Gy irradiated, no drug treatment) is not necessary. In comparison, the relation between tirapazamine toxicity and oxygen tension changes continually over a wide range of oxygen concentrations (Koch, 1993), making it difficult to distinguish the significant hypoxic fraction. A second advantage relates to the rate of repair of DNA damage by these bioreductive drugs. While half of the tirapazimine breaks were rejoined within 1 h after drug treatment, no repair of RSU 1069 cross-links was seen for up to 4 h following drug treatment, providing a much longer sample window and simplifying interpretation of results. A third benefit is that irradiation of cells (once extracted from the tumour) greatly enhances the ability to detect hypoxic cells containing RSU 1069 cross-links, thus reducing the amount of drug required. While the gastrointestinal toxicity of RSU 1069 precludes the use of this drug in the clinic, RB 6145 and its isomer PD 144872 are less emetic compounds which act as prodrugs for RSU 1069, and will advance to clinical trial (Cole *et al.*, 1992; Sebolt-Leopold *et al.*, 1992; Bremner, 1993). Since RB 6145 is converted to RSU 1069 with a half-time of less than 2 min at 37°C (Binger and Workman, 1991), we can be assured that results with RSU 1069 will also apply to the prodrug chosen for clinical testing.

A final consideration in comparing tirapazamine and RSU 1069 as indicators of hypoxia is that hypoxic cells show less DNA damage than aerobic cells after RSU 1069 treatment, but more damage than aerobic cells after tirapazamine exposure. The fact that the cells of interest, the hypoxic cells, show a smaller tail moment after RSU 1069 can be advantageous since some tumours (especially during therapy) may accumulate significant numbers of heavily damaged cells which might be mistakenly considered hypoxic following treatment with tirapazamine.

The kinetics of strand break and cross-link repair was somewhat unusual in that cross-link damage was stable, while strand breaks appeared to show a delay or even an increase in damage prior to rejoining (Figure 4). These kinetics can be compared with those obtained by Jenner *et al.* (1991) using alkaline or neutral sucrose gradient sedimentation to detect single- or double-strand breaks in V79 cells exposed to much higher doses (0.4 to 2 mM RSU 1069). Their results indicate little or no rejoining of single-strand breaks over a 3 h period, and an actual increase in the number of double-strand breaks during this period. While the chemical nature of the DNA lesions being evaluated by the comet assay may differ, it is clear from both these studies that damage by RSU 1069 is not easily repaired.

Like tirapazamine, the difference in response to RSU 1069 between poorly perfused and well-perfused cells is impressive. Hoechst 33342 injection prior to sorting stains those cells close to functional blood vessels at that point in time, since the plasma half-life of this drug is less than 2 min (Chaplin *et al.*, 1987). However, both tirapazamine and RSU 1069 have plasma half-lives of about 20–30 min (Workman and Walton, 1984; Walton and Workman, 1993); as cells cycle in and out of hypoxia, they can still generate active species that cause DNA damage. For a similar reason, infusion of Hoechst during irradiation significantly improved the ability of the sorting method to detect cells resistant to killing by X-rays (Chaplin *et al.*, 1987).

In summary, RSU 1069 produces DNA cross-links in hypoxic cells that can be detected using the alkaline comet assay. Irradiation of the single-cell suspension obtained from tumours after drug treatment provides additional sensitivity for detecting cells with cross-links. There appears to be good potential for the application of this method to detect hypoxic cells in human tumours exposed to the less emetic prodrug, PD144872.

**Acknowledgements**

This work was supported by Grant No. CA 37879 awarded by the National Cancer Institute DHHS. The author gratefully acknowledges the expert technical assistance of Charlene M Vikse.

**References**

BINGER M AND WORKMAN P. (1991). Pharmacokinetic contribution to the improved therapeutic selectivity of a novel bromoethylamino prodrug (RB 6145) of the mixed-function hypoxic cell sensitizer/cytotoxin α-(1-aziridinomethyl)-2-nitro-1H-imidazole-1-ethanol (RSU 1069). *Cancer Chemother. Pharmacol.*, **29**, 37–47.

BREMNER JC. (1993). Assessing the bioreductive effectiveness of the nitroimidazole RSU 1069 and its prodrug RB 6145 with particular reference to in vivo methods of evaluation. *Cancer Metastasis. Rev.*, **12**, 177–193.

CHAPLIN DJ, DURAND RE AND OLIVE PL. (1985). Cell selection from a murine tumour using the fluorescent probe Hoechst 33342. *Br. J. Cancer*, **51**, 569–572.

CHAPLIN DJ, OLIVE PL AND DURAND RE. (1987). Intermittent blood flow in a murine tumour: radiobiological effects. *Cancer Res.*, **47**, 597–601.

COLE S, STRATFORD IJ, FIELDEN EM, ADAMS GE, LEOPOLD W, ELLIOTT W, SUTO M AND SEBOLT-LEOPOLD J. (1992). Dual function nitroimidazoles less toxic than RSU 1069: selection of candidate drugs for clinical trial (RB 6145 and/or PD 130908). *Int. J. Radiat. Oncol. Biol. Phys.*, **22**, 545–548.

CRUMP PW, FIELDEN EM, JENNER TJ AND O'NEILL P. (1990). A comparison of the techniques of alkaline filter elution and alkaline sucrose sedimentation used to assess DNA damage induced by 2-nitroimidazoles. *Bicohem. Pharmacol.*, **40**, 621–627.

DURAN RE AND OLIVE PL. (1992). Evaluation of bioreductive drugs in multicell systems. *Int. J. Radiat. Oncol. Biol. Phys.*, **22**, 689–692.

HILL RP, GULYAS S AND WHITMORE GF. (1986). Studies of the in vivo and in vitro cytotoxicity of the drug RSU 1069. *Br. J. Cancer*, **53**, 743–751.

HILL RP, GULYAS S AND WHITMORE GF. (1989). Toxicity of RSU-1069 for KHT cells treated in vivo or in vitro: evidence for a diffusible toxic product. *Int. J. Radiat. Oncol. Biol. Phys.*, **16**, 1111–1114.

JENNER TJ, O'NEILL P, CRUMP PW, FIELDEN EM, SAPORA O AND SANTODANATO L. (1991). The repair of DNA damage induced in V79 mammalian cells by the nitroimiazole aziridine, RSU 1069. *Biochem. Pharmacol.*, **42**, 1711–1704.

KOCH CJ. (1993). Unusual oxygen concentration dependence of toxicity of SR-4233, a hypoxic cell toxin. *Cancer Res.*, **53**, 3992–3997.

OLIVE PL. (1994). Radiation-induced reoxygenation in the SCCVII murine tumour: evidence for a decrease in oxygen consumption and an increase in tumour perfusion. *Radiother. Oncol.*, **32**, 37–46.

OLIVE PL AND BANÁTH JP. (1993). Induction and rejoining of radiation-induced DNA single-strand breaks: tail moment as a function of position in the cell cycle. *Mutat. Res.*, **294**, 275–283.

OLIVE PL AND DURAND RE. (1992). Detecting hypoxic cells in a murine tumour using the comet assay. *J. Natl Cancer Inst.*, **85**, 707–711.

OLIVE PL, DURAND RE AND CHAPLIN DC. (1987). Cytotoxicity of RSU 1069 in spheroids and murine tumours. *Int. J. Radiat. Oncol. Biol. Phys.*, **13**, 1361–1366.

OLIVE PL, DURAND RE, LE RICHE J, OLIVOTTO I AND JACKSON SM. (1993). Gel electrophoresis of individual cells to quantify hypoxic fraction in human breast cancers. *Cancer Res.*, **53**, 733–736.

O'NEILL P, MCNEIL SS, JENKINS TC. (1987). Induction of DNA crosslinks in vitro upon reduction of the nitroimidazole-aziridines RSU 1069 and RSU-1131. *Biochem. Pharmacol.*, **36**, 1787–1792.

SEBOLT-LEOPOLD S, VINCENT PW, BENINGO KA, ELLIOTT WL, LEOPOLD WR, HEFFNER TG, WILEY JN, STIER MA AND SUTO MJ. (1992). Pharamcologic/pharmacokinetic evaluation of emesis induced by analogs of RSU 1069 and its control by antiemetic agents. *Int. J. Radiat. Oncol. Biol. Phys.*, **22**, 549–551.

SILVER ARJ, O'NEIL P AND JENKINS TC. (1985). Induction of DNA strand breaks by RSU 1069, a nitroimiazole-aziridine radiosensitizer. *Biochem. Pharmacol.*, **34**, 3537–3542.

STONE HB, BROWN JM, PHILLIPS TL AND SUTHERLAND RM. (1993). Oxygen in human tumours: correlations between methods of measurement and response to therapy. *Radiat. Res.*, **136**, 422–434.

STRATFORD IJ, O'NEILL P, SHELDON PW, SILVER ARJ, WALLING JM AND ADAMS GE. (1986*a*). RSU 1069, a nitroimidazole containing an aziridine group: bioreduction greatly increases cytotoxicity under hypoxic conditions. *Biochem. Pharmacol.*, **35**, 105–110.

STRATFORD IJ, WALLING JM AND SILVER ARJ. (1986*b*). The differential cytotoxicity of RSU 1069 – cell survival studies indicating interaction with DNA as a possible mode of action. *Br. J. Cancer*, **53**, 339–344.

WALTON MI AND WORKMAN P. (1993). Pharmacokinetics and bioreductive metabolism of the novel benzotriazine di-N-oxide hypoxic cell cytotoxin WIN 59075 (SR 4233: NCS 130181) in mice. *J. Pharmacol. Exp Ther.*, **265**, 938–947.

WHITMORE GF AND GULYAS S. (1986). Studies on the toxicity of RSU 1069. *Inter. J. Radiat. Oncol. Biol. Phys.*, **12**, 1219–1222.

WORKMAN P AND WALTON MI. (1984). Pharmacology of the mixed function radio- and chemosensitizers CB 1954 and RSU 1069. *Int. J. Radiat. Oncol. Biol. Phys.*, **10**, 1307–1310.